# Supplementary material for: Cross-site transportability of an explainable artificial intelligence model for acute kidney injury prediction
Source: Nat Commun. 2020 Nov 9;11:5668. doi: 10.1038/s41467-020-19551-w (PMC7653032; doi:10.1038/s41467-020-19551-w)
Supplement: Supplementary file 1 — Supplementary Information [file 41467_2020_19551_MOESM1_ESM.pdf]

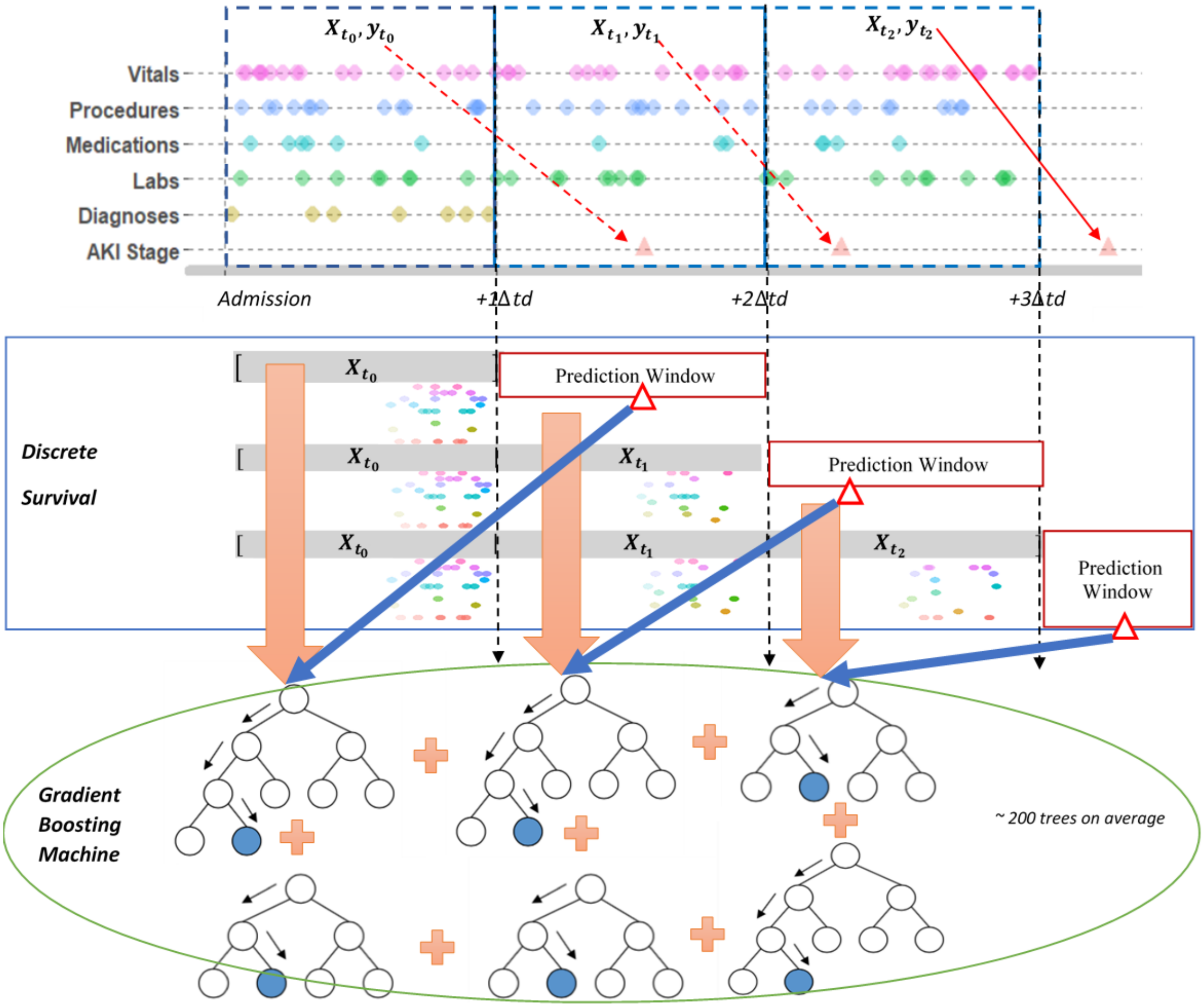

Supplemental Figure 1. Illustration of Discrete-time Survival Gradient Boosted Tree Model. Different colored circles represent different types of clinical data. Red triangles represent real values of the outcome (i.e., AKI stage in the following prediction window). X_{t_i} denotes all available clinical features collected strictly before time t_i (i.e., day since admission), while y_{t_i} denotes AKI stage within the prediction window. All electronic health records of each patient were structured as a group of observations occurring within different discrete time intervals.

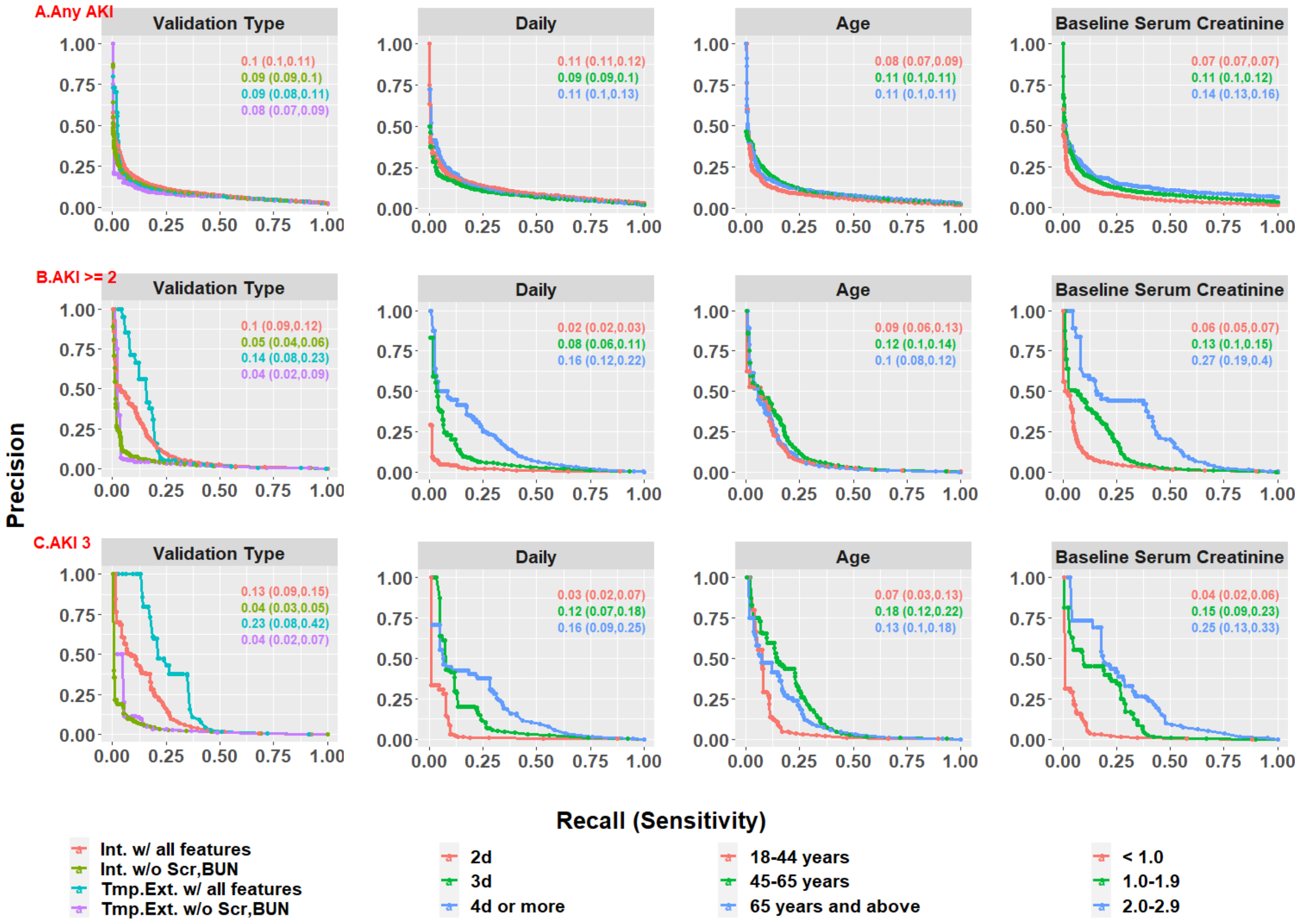

Supplemental Figure 2. DS-GBT Model performance on the source health system data illustrated by precision-recall curves for predicting AKI events of any severity (**a**), at least stage 2 (**b**), or stage 3 (**c**) within the next 48-hours for various subgroups.

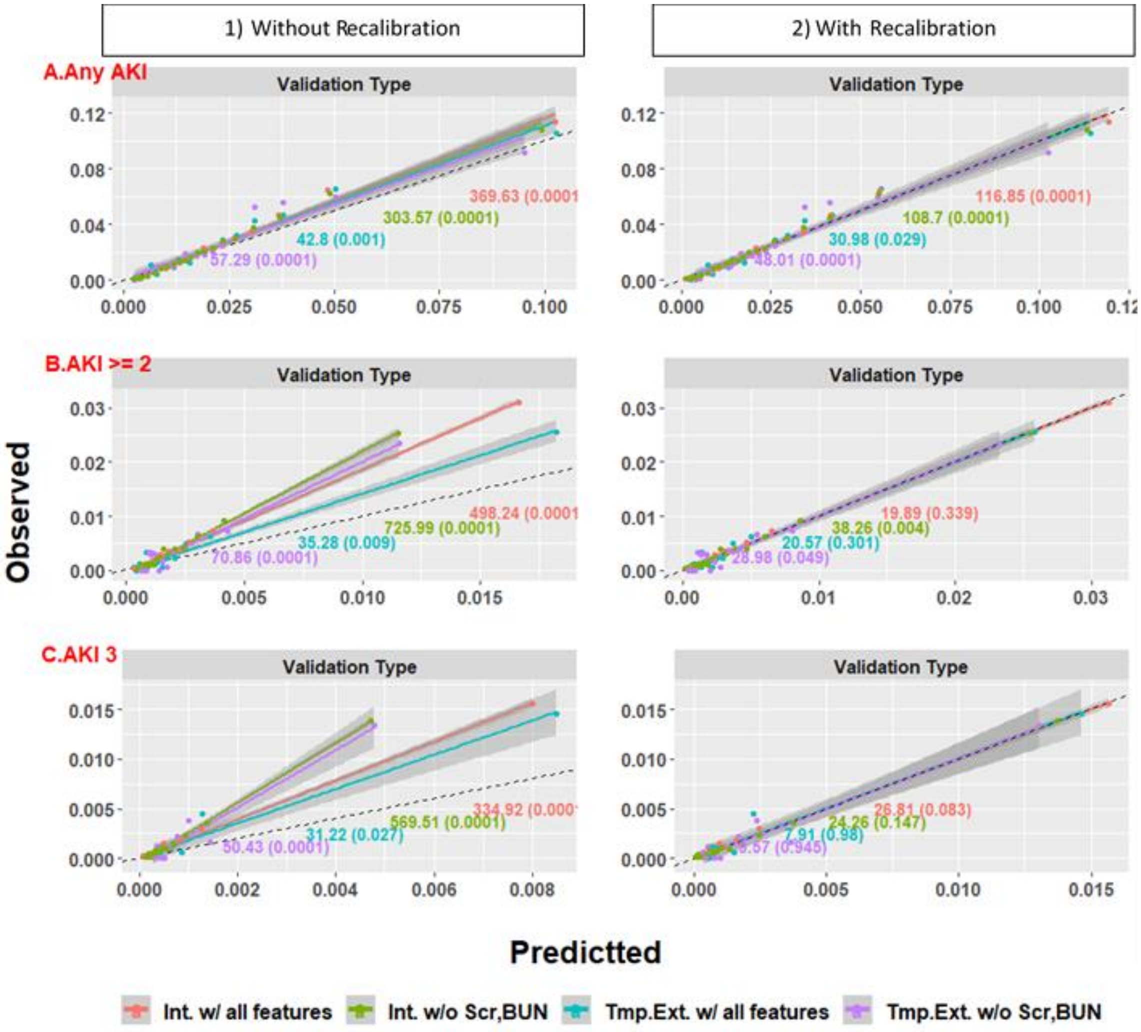

Supplemental Figure 3. DS-GBT Model calibrations based on source health system data for predicting AKI events of any severity (**a**), at least stage 2 (**b**), or stage 3 (**c**) within the next 48-hours for various subgroups, before (left) and after (right) recalibrations using isotonic regression. Chi-square scores and P-values are also reported. 95% confidence band for each calibration line is shown as the shaded area in each figure.

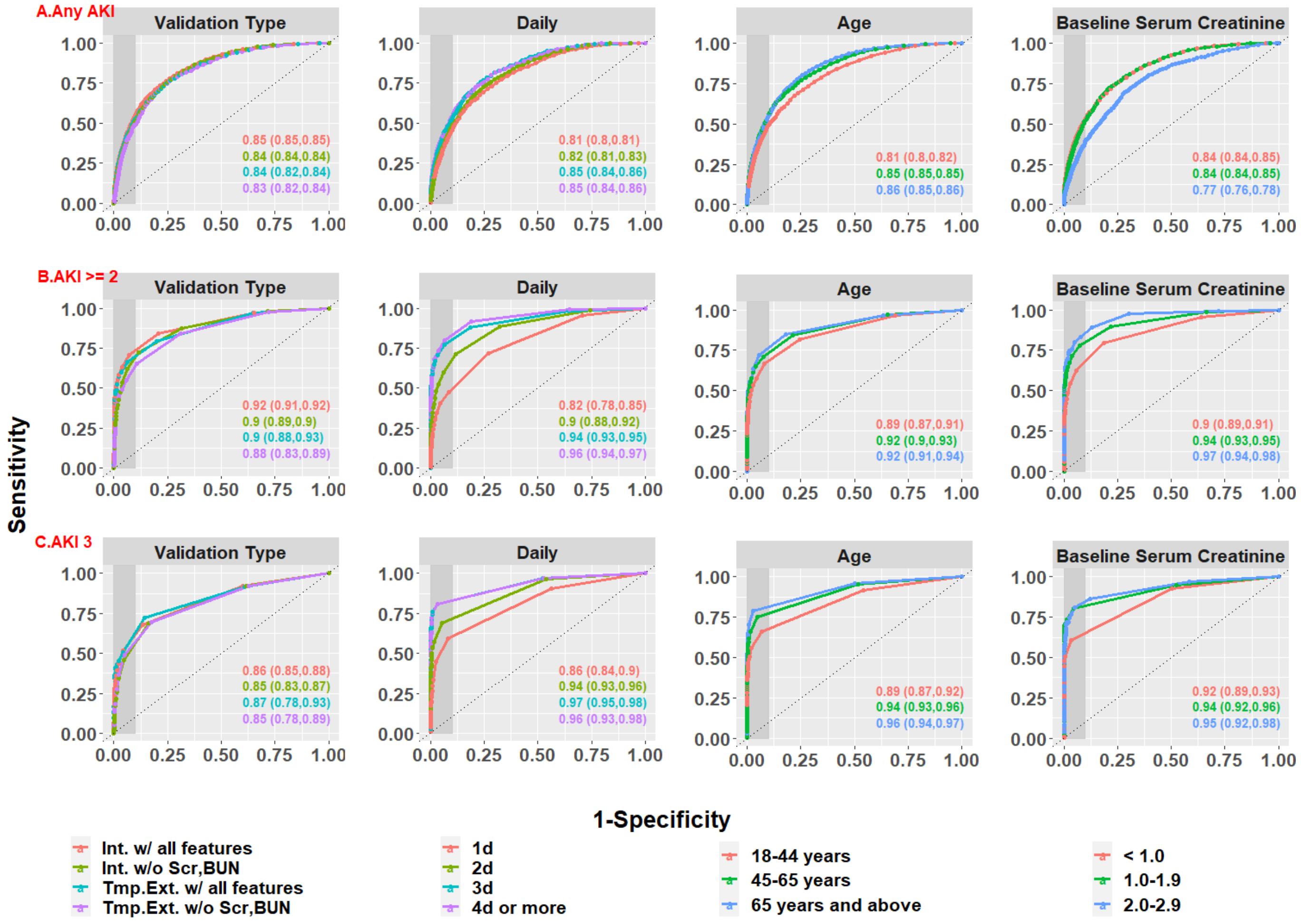

Supplemental Figure 4. DS-GBT Model performance on the source health system data illustrated by receiver operating characteristic curves for predicting AKI events of any severity (**a**), at least stage 2 (**b**), or stage 3 (**c**) within the next 24-hours for various subgroups.

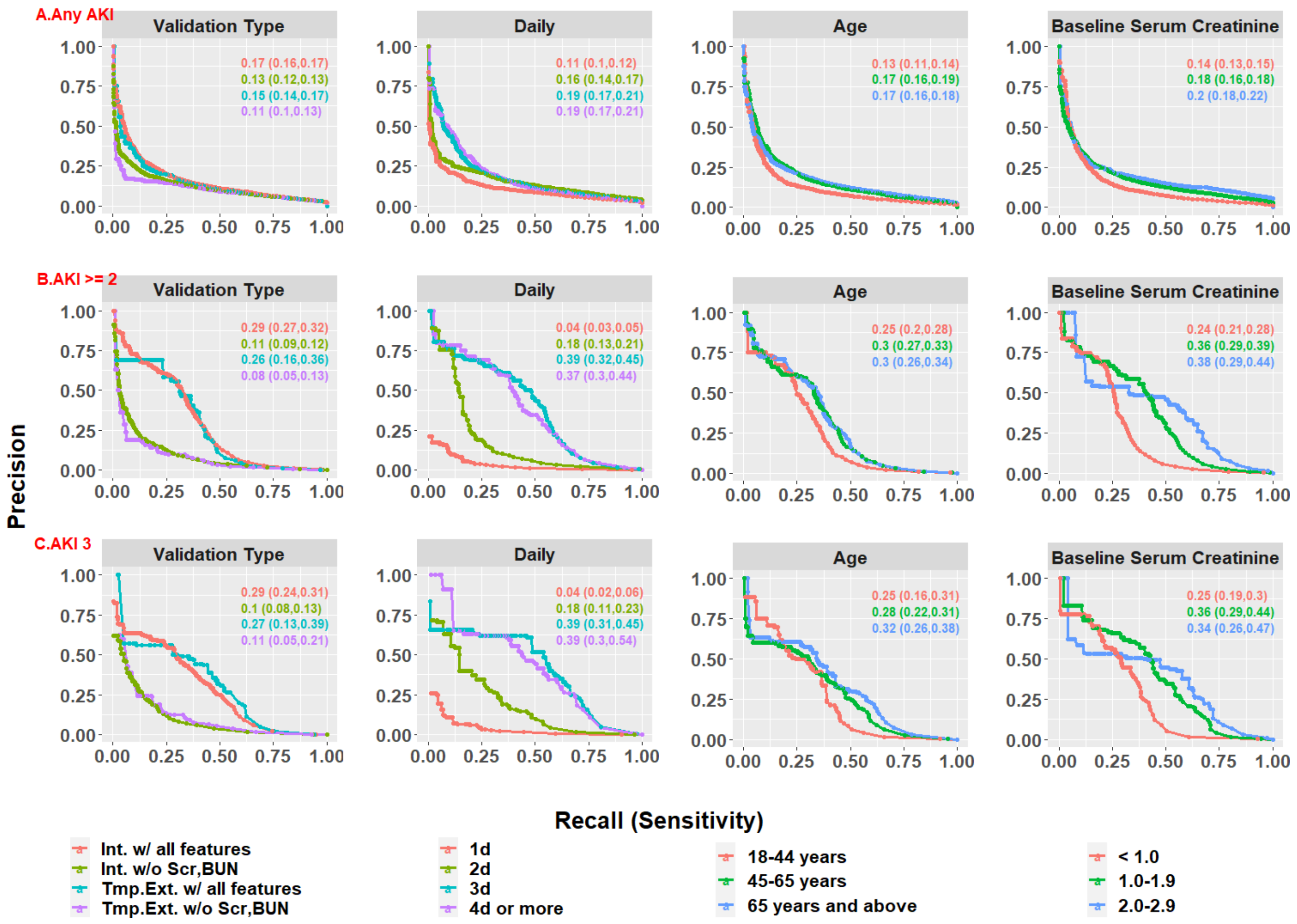

Supplemental Figure 5. DS-GBT Model performance on the source health system data illustrated by precision recall curves for predicting AKI events of any severity (**a**), at least stage 2 (**b**), or stage 3 (**c**) within the next 24-hours for various subgroups.

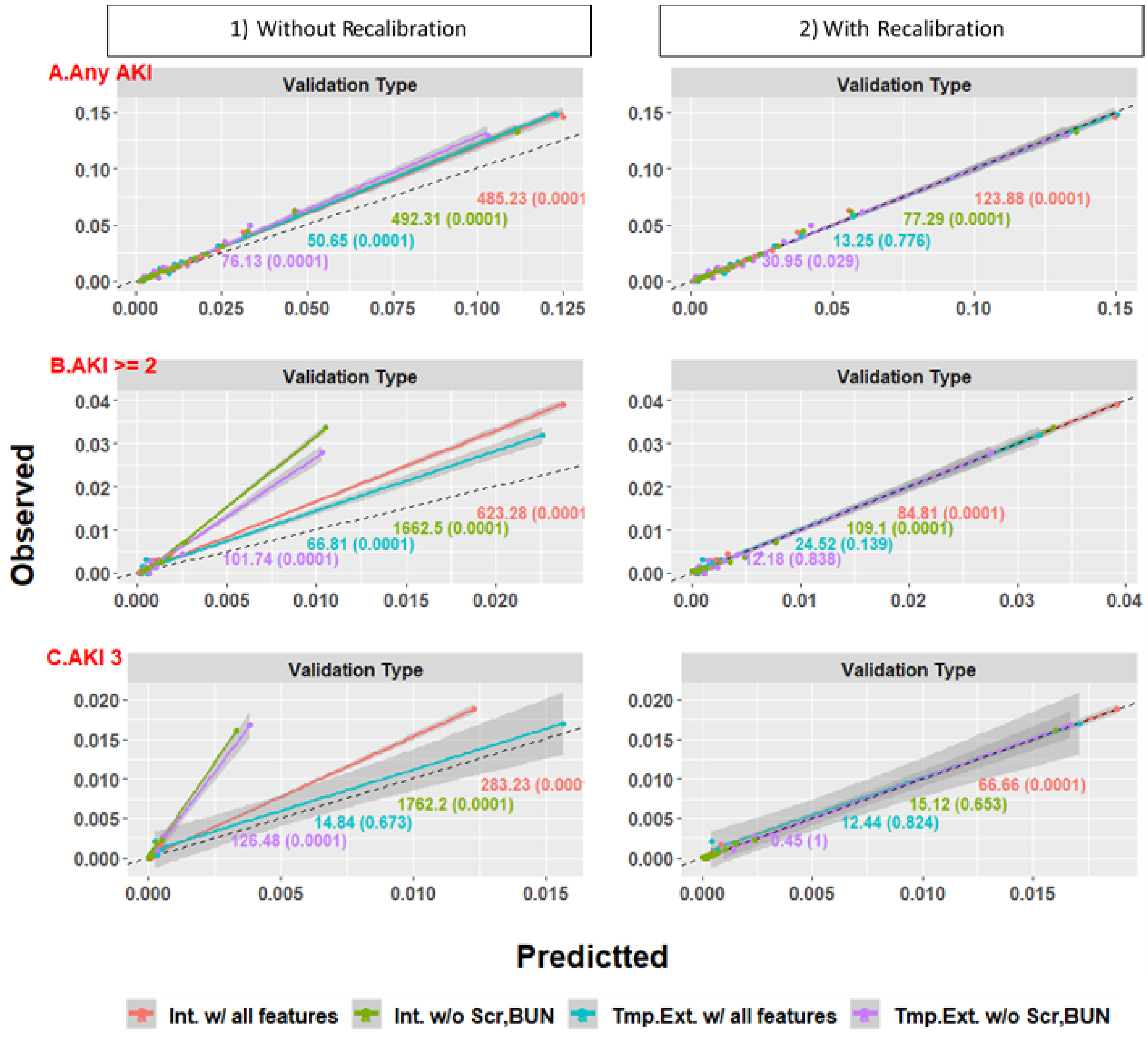

Supplemental Figure 6. DS-GBT Model calibrations based on source health system data for predicting AKI events of any severity (**a**), at least stage 2 (**b**), or stage 3 (**c**) within the next 24-hours for various subgroups, before (left) and after (right) recalibrations using isotonic regression. Chi-square scores and P-values are reported. 95% confidence band for each calibration line is shown as the shaded area in each figure.

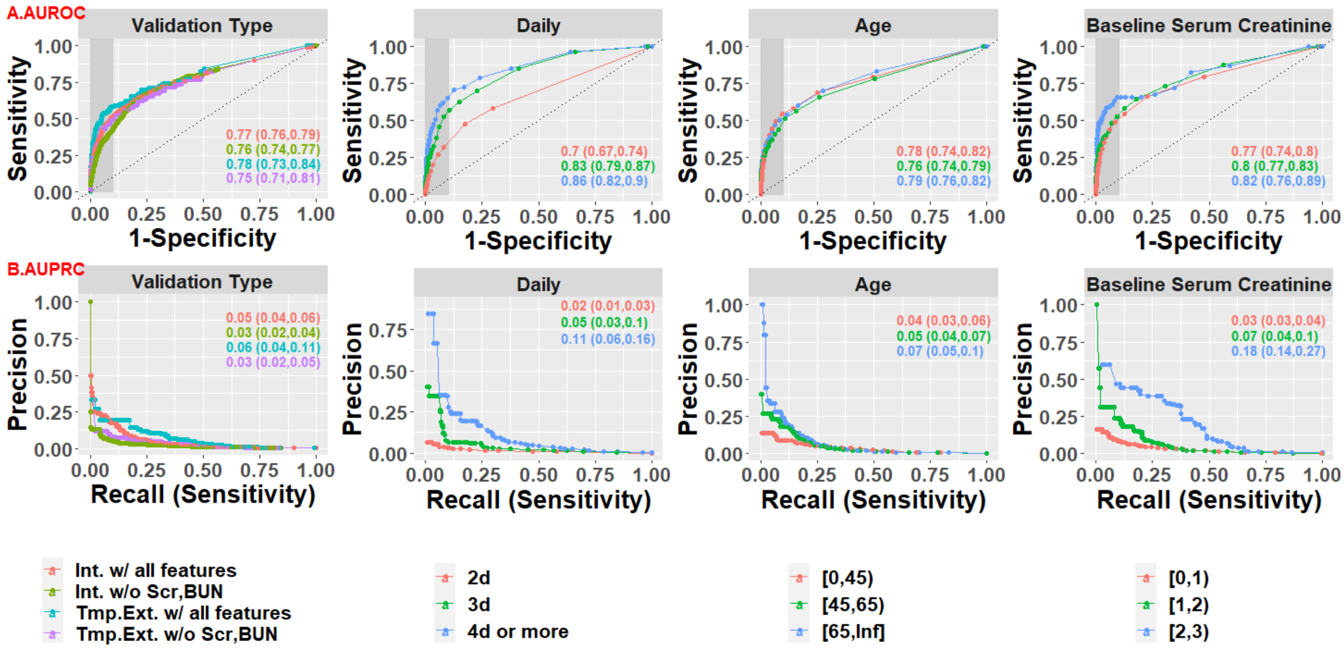

Supplemental Figure 7. LASSO Model performance on the source health system data illustrated by receiver operating characteristic curves and precision recall curves for predicting AKI events of at least stage within the next 48-hours for various subgroups.

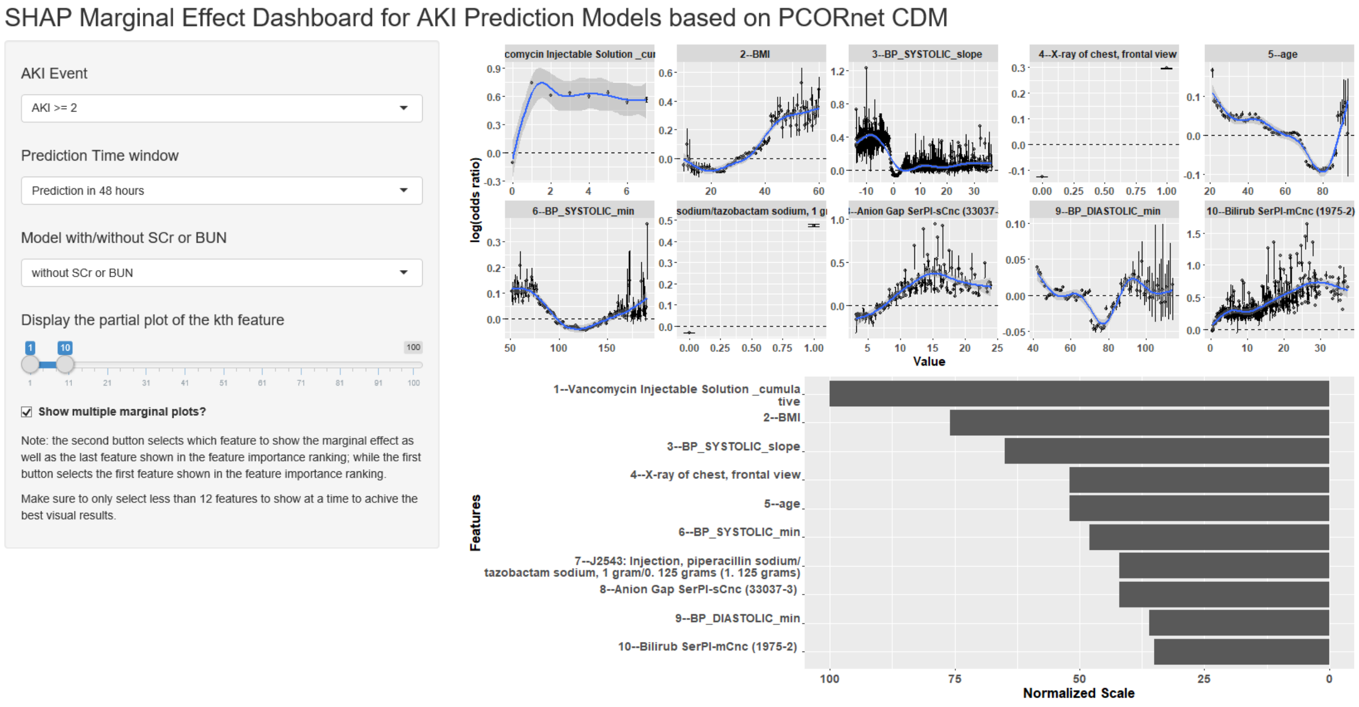

Supplemental Figure 8. Marginal plots of the top 10 important variables for predicting moderate to severe AKI (at least AKI stage 2) in 48 hours. Each panel demonstrates marginal effects of one of the most impactful features ranked among top 10 by the model without SCr and BUN for predicting moderate-to-severe AKI in 48 hours. Each dot represents an average change of odds ratio for a variable, taking certain values within a bootstrapped sample. Each error bar depicts a 95% bootstrap confidence interval based on 100 bootstrapped samples. The dashed horizontal line shows an odds ratio of 1. The 'shaded area' represents the 95% confidence band for the lowess smoother extrapolating across all dots. The full interactive dashboard can be found at: https://sxinger.shinyapps.io/AKI_shap_dashbd/

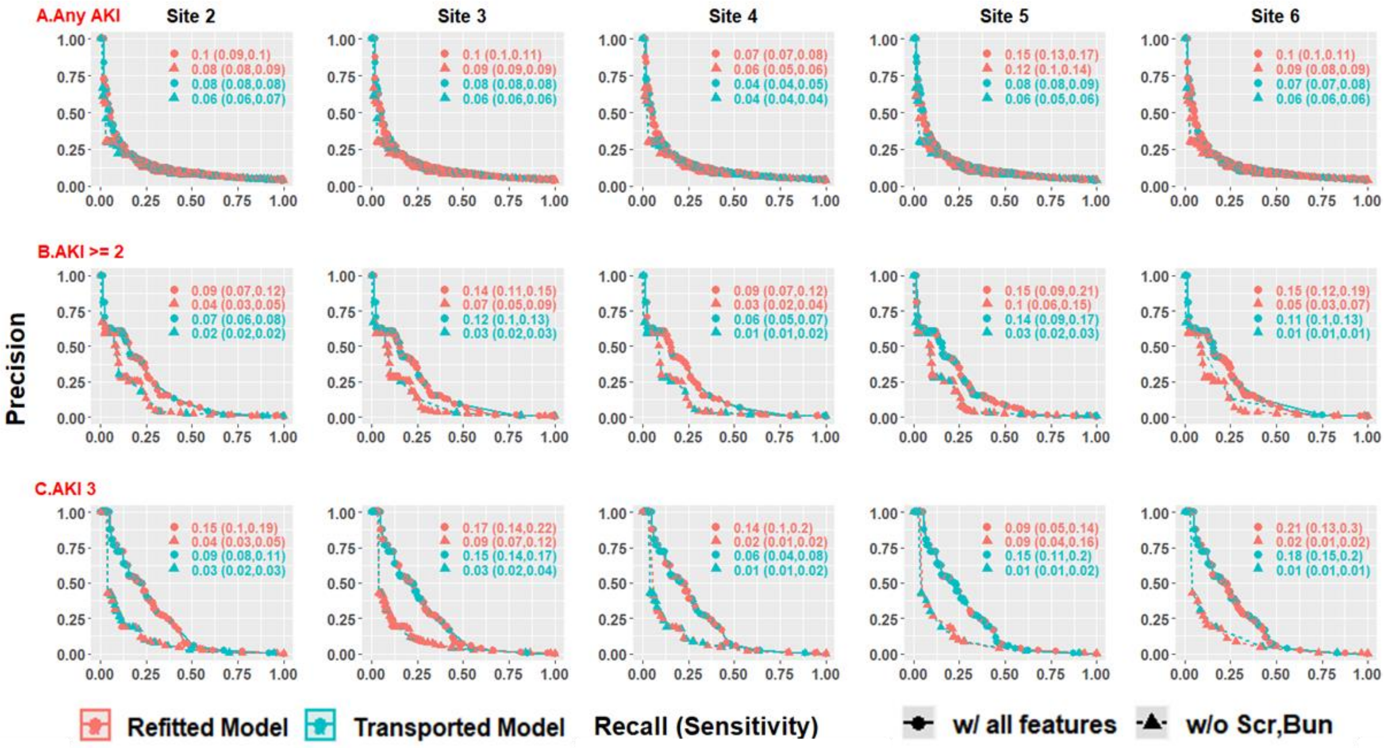

Supplemental Figure 9. Comparison of DS-GBT model performance illustrated by precision recall curves for transported model vs refitted model in 24-hour AKI predictions on data from external validation sites.

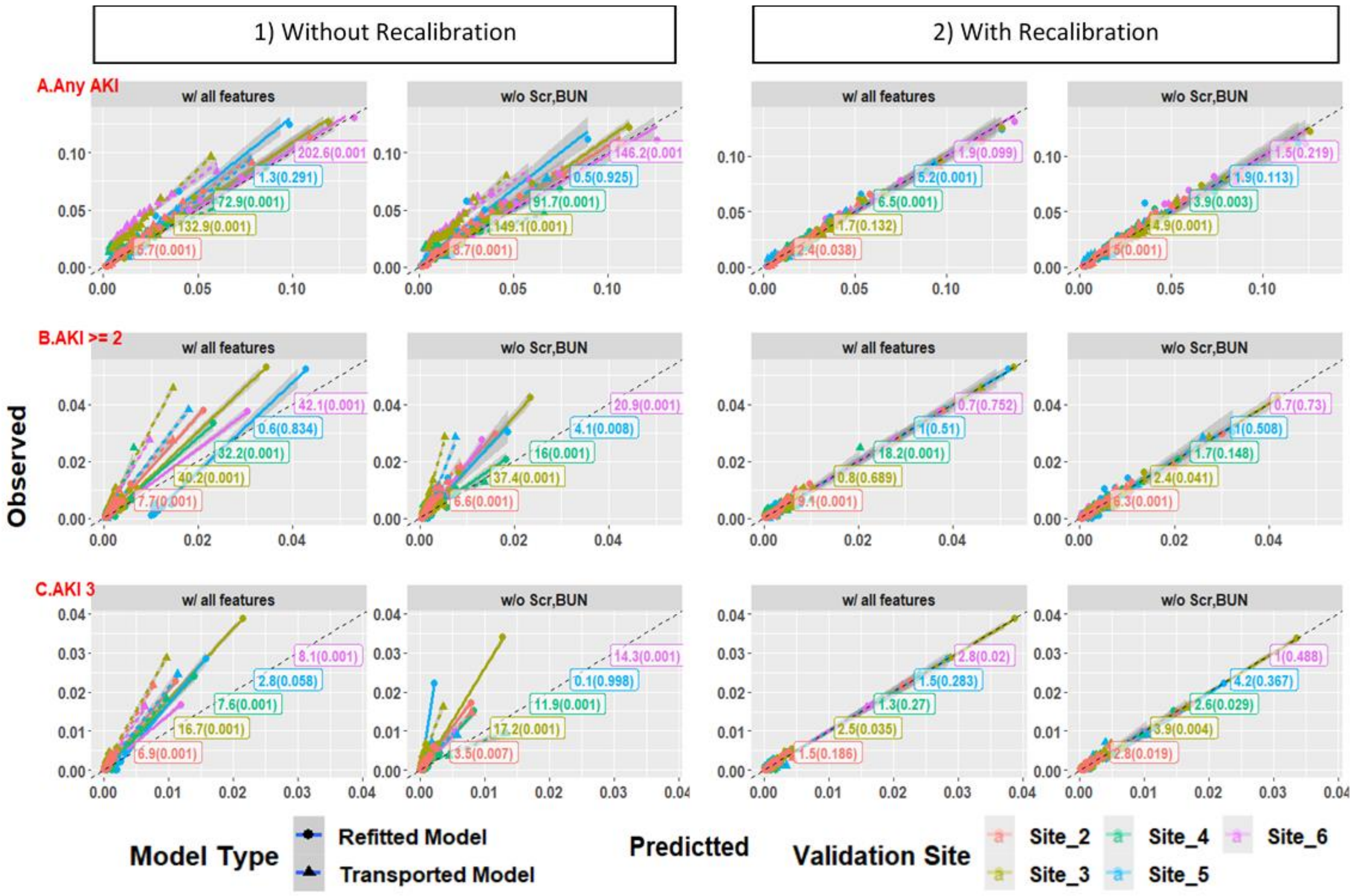

Supplemental Figure 10. DS-GBT Model calibration comparison for target health system data for predicting AKI events of any severity (**a**), at least stage 2 (**b**), or stage 3 (**c**) within the next 48-hours for various subgroups, before (left) and after (right) recalibrations using isotonic regression. F-scores and P-values comparing refitted model calibrations with transferred models are reported. Each F-score was calculated as the ratio of Hosmer-Lemeshow (HL) Chi-squared scores between refitted and transported model, while the P-value was based on two-sided F-test.

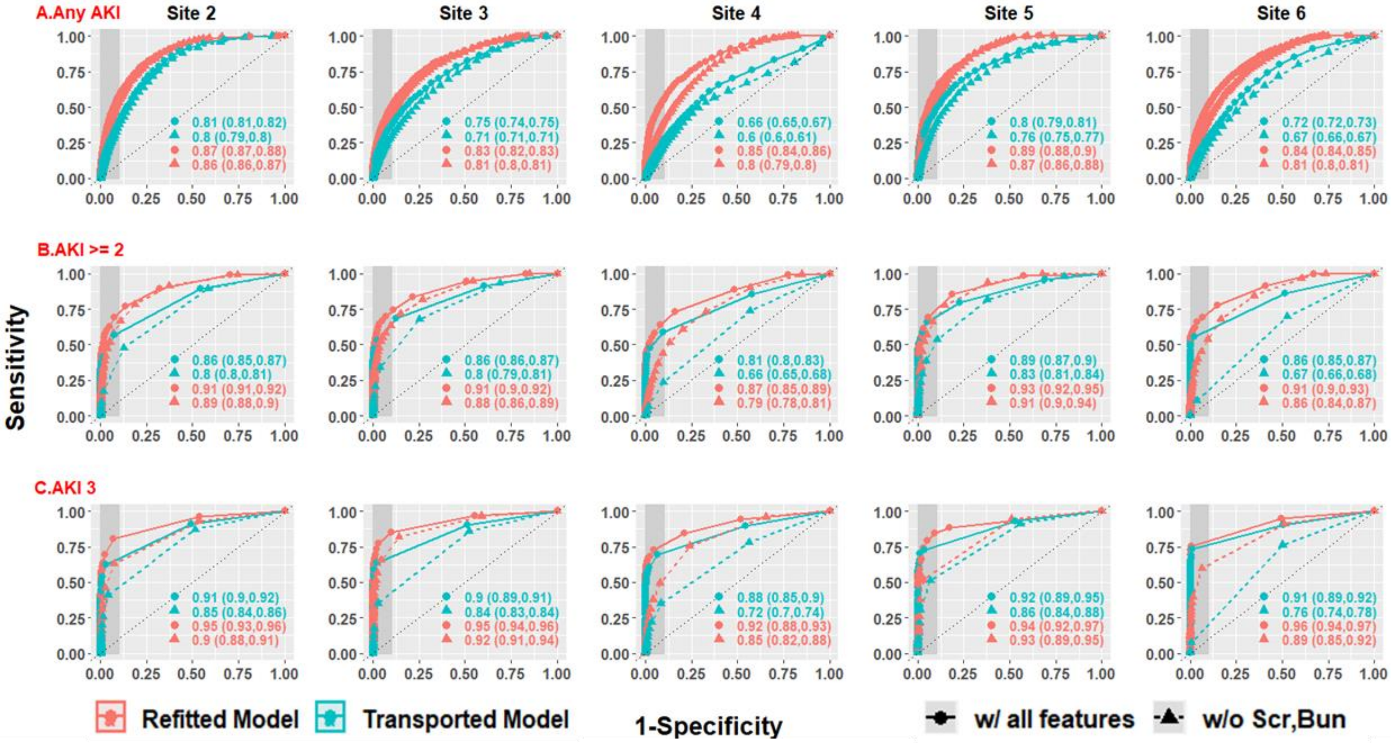

Supplemental Figure 11. Comparison of DS-GBT model performance illustrated by receiver operating characteristic curves for transported model vs refitted model in 24-hour AKI prediction on external validation site data.

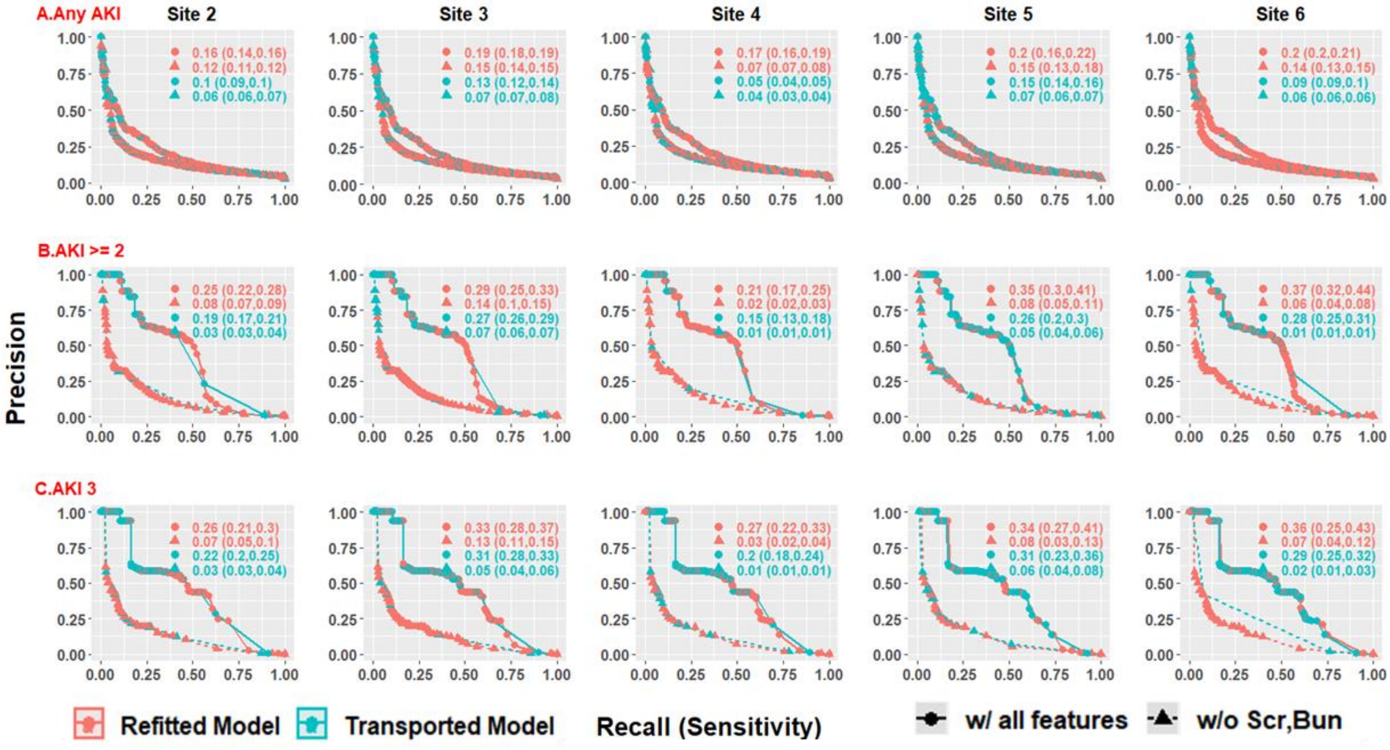

Supplemental Figure 12. Comparison of DS-GBT model performance illustrated by precision recall curves for transported model vs refitted model in 24-hour AKI prediction on data from external validation sites.

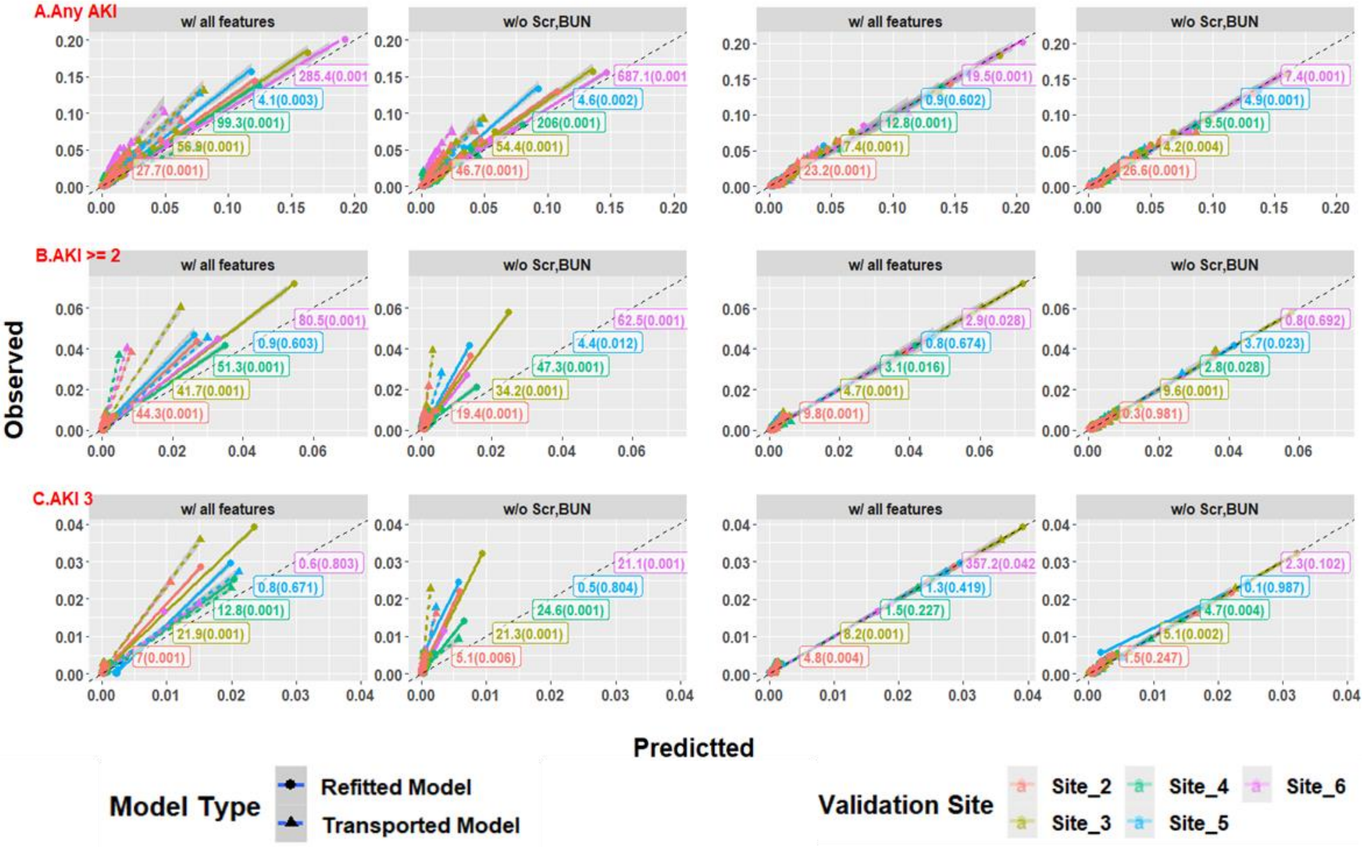

Supplemental Figure 13. DS-GBT Model calibration comparison for target health system data for predicting AKI events of any severity (**a**), at least stage 2 (**b**), or stage 3 (**c**) within the next 24-hours for various subgroups, before (left) and after (right) recalibrations using isotonic regression. F-scores and P-values comparing refitted model calibrations with transferred models are reported. Each F-score was calculated as the ratio of Hosmer-Lemeshow (HL) Chi-squared scores between refitted and transported model, while the P-value was based on two-sided F-test.

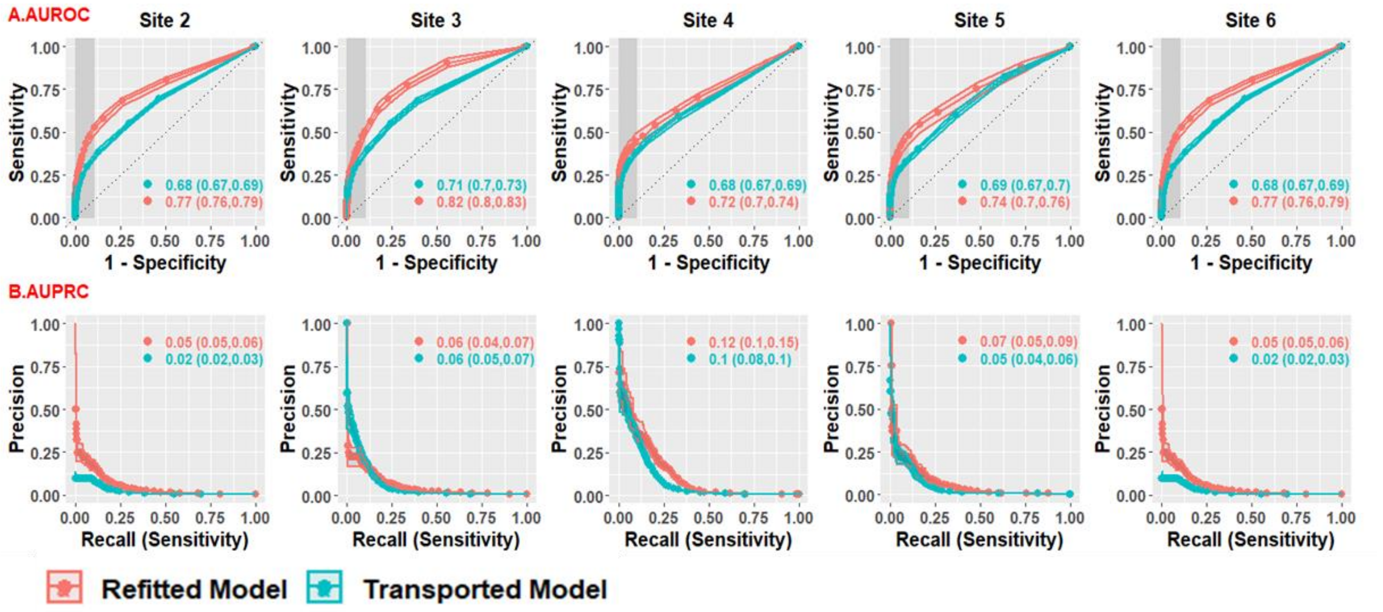

Supplemental Figure 14. Comparison of LASSO model performance illustrated by receiver operating characteristic curves and precision recall curves for transported model vs refitted model in 48-hour AKI prediction on data from external validation sites.

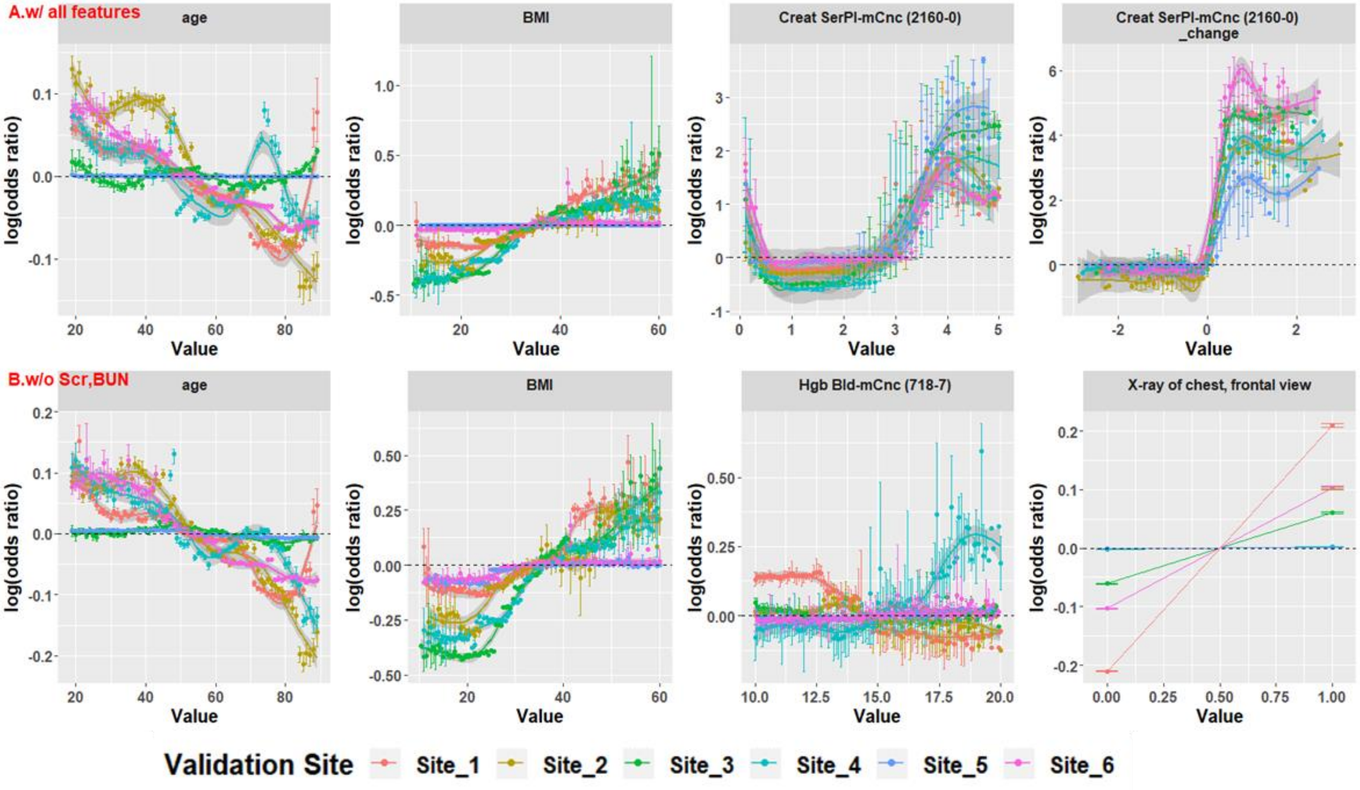

Supplemental Figure 15. Marginal effects difference of top important variables for predicting moderate to severe AKI (at least AKI stage 2) in 48 hours. Marginal effects are measured by SHAP value of top four variables that were deemed important in both the source health system and all five target health systems (based on Figure 4). Each dot represents an average change of odds ratio for a variable, taking certain values within a bootstrapped sample. Each colored vertical line depicts a 95% confidence interval based on 100 bootstrapped samples. The dashed horizontal line shows an odds ratio of 1.

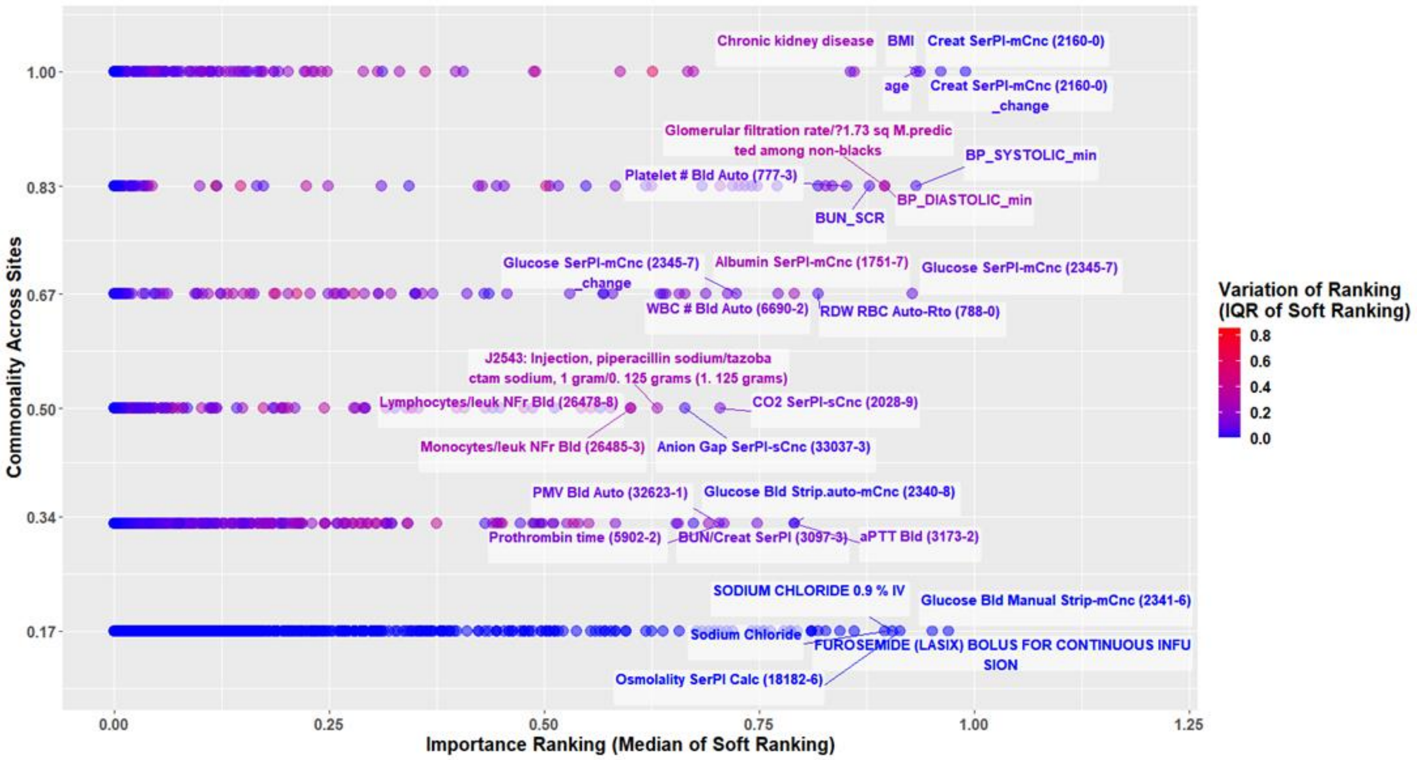

Supplemental Figure 16. Feature Selection Disparities Across Sites (48-hour predictions for any AKI). The figure demonstrates feature importance disparities for the models trained on source data as well as refitted models at each validation site, using all features. Each dot corresponds to one of the most important features ranked among the top-100 by at least one of the six models; y-axis measures the proportions of sites that identified the feature as top-100, or "commonality across sites"; x-axis measures the median of variable importance rankings measured as "soft ranking" (the closer it is to 1, the higher the feature ranks), which is also color coded by the interquartile range (IQR) of the ranks across sites (the higher the IQR is, the more disagreement across sites on the importance of that feature).

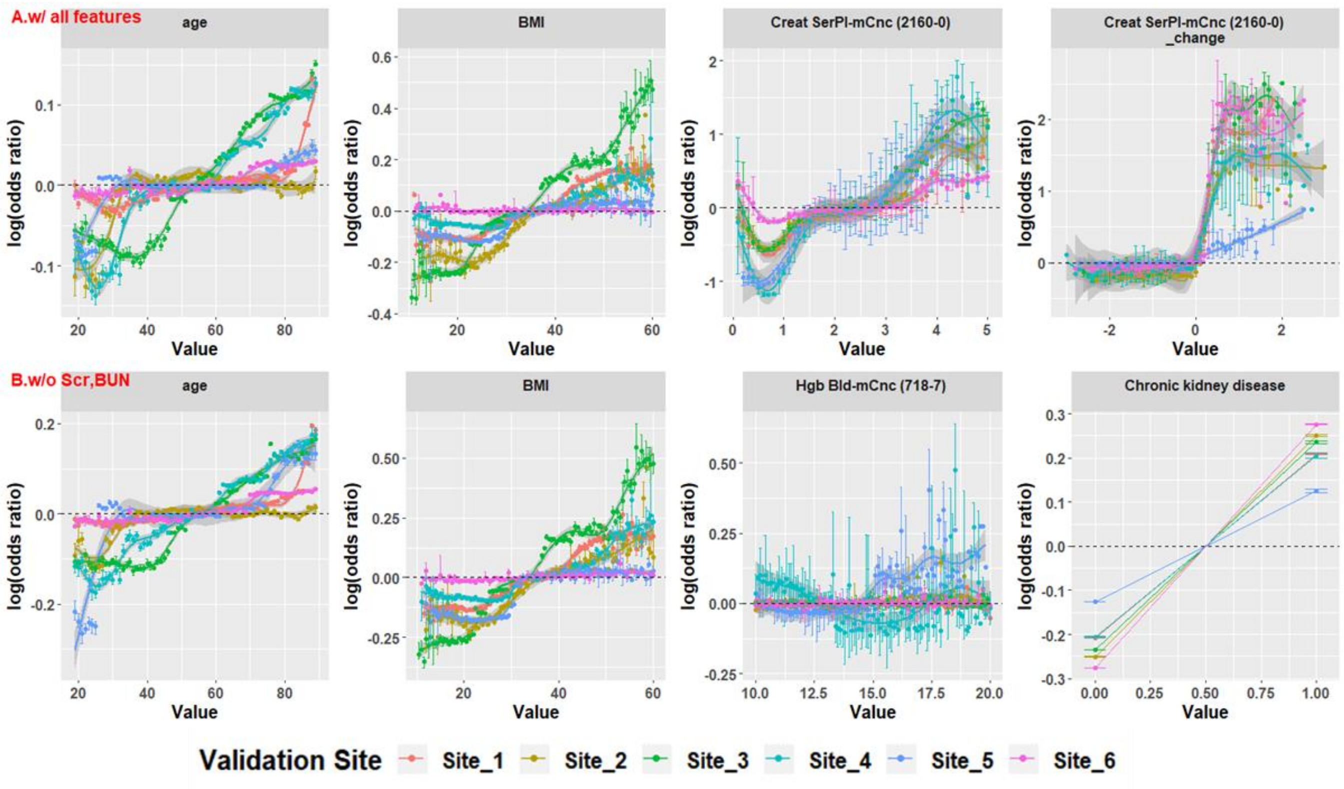

Supplemental Figure 17. Marginal effects difference of top important variables for predicting any AKI in 48 hours. Marginal effects are measured by SHAP value of top four variables that commonly presents in both the source system and all the five target systems (based on Extended Figure 8). Each dot represents an average change of odds ratio for a variable taking certain values within a bootstrapped sample. Each colored vertical line depicts a 95% confidence interval based on 100 bootstrapped samples. The dashed horizontal line corresponds to a reference odds ratio of 1.

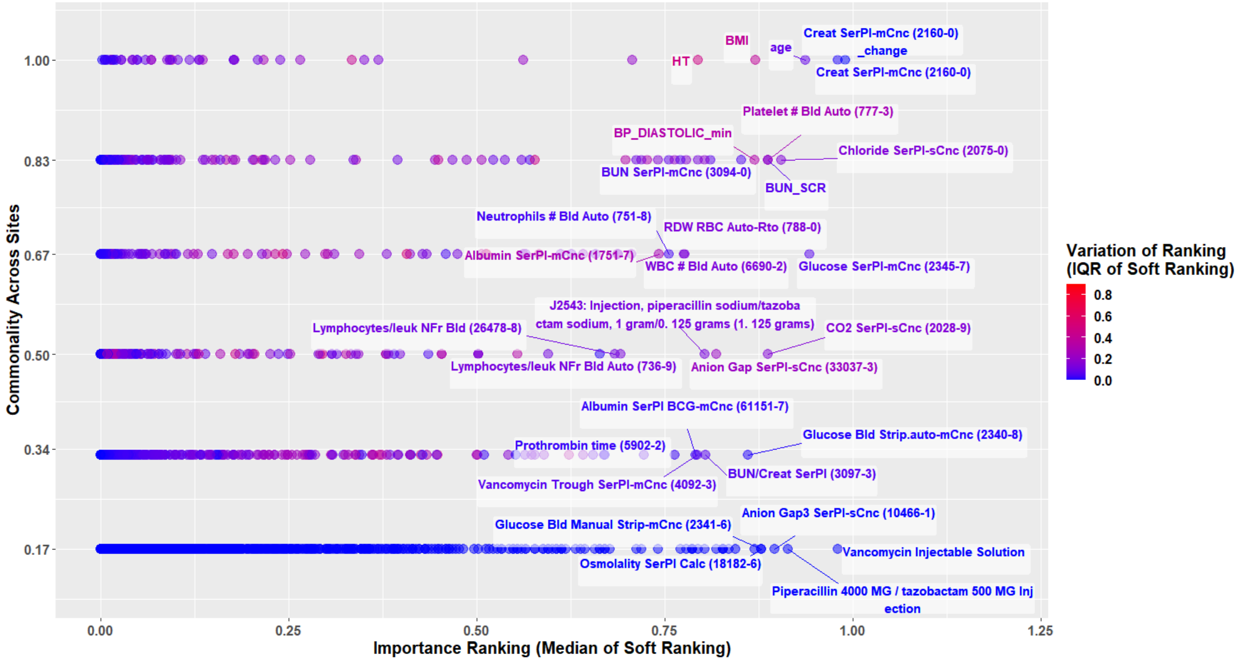

Supplemental Figure 18. Feature Selection Disparities Across Sites (48-hour predictions for moderate-to-severe AKI). The figure demonstrates feature importance disparities for the models trained on source data as well as refitted models at each validation site, with SCr and BUN removed.

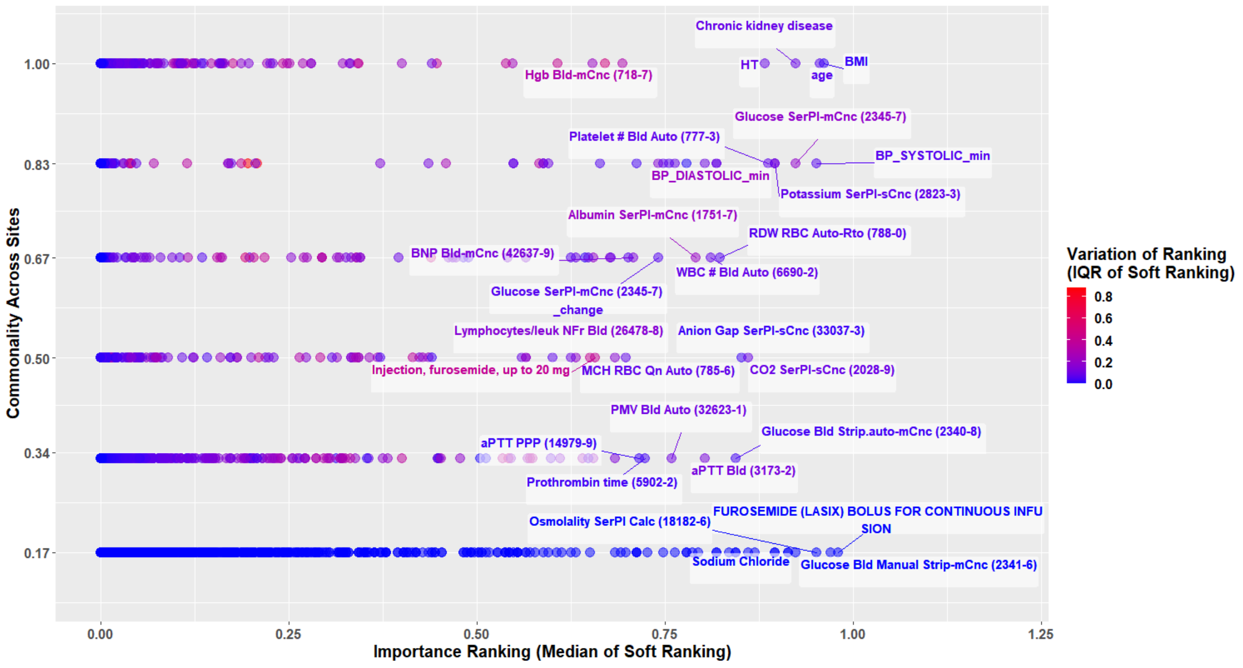

Supplemental Figure 19. Feature Selection Disparities Across Sites (48-hour predictions for any AKI). The figure demonstrates feature importance disparities for the models trained on source data as well as refitted models at each validation site, with SCr and BUN removed.

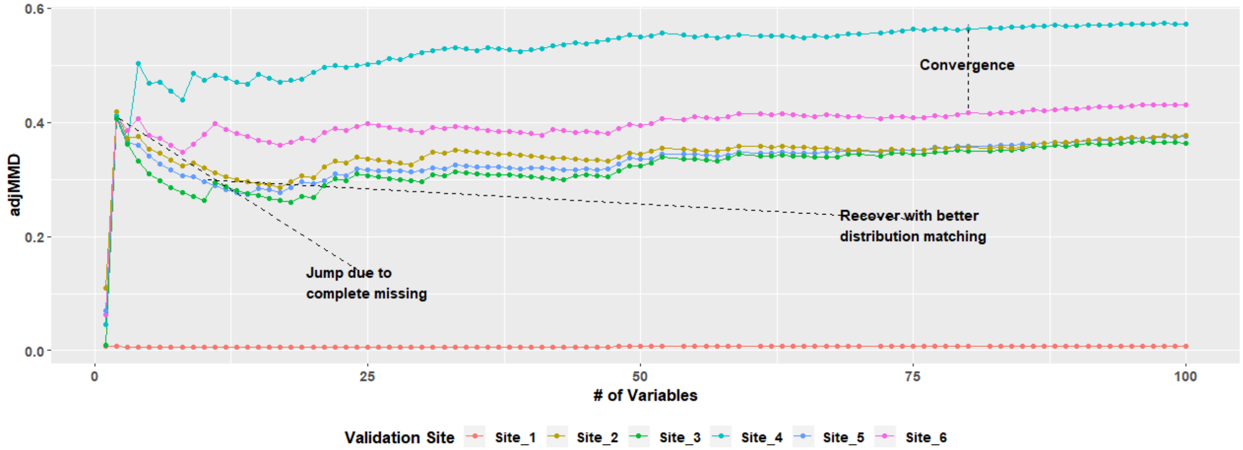

Supplemental Figure 20. Relationship between adjMMD and number of variables for model of 48-hour for moderate-to-severe AKI predictions using all features, which shows the expected behaviors of adjMMD and identifies the top 13 variables to be a sufficient sets to produce effective adjMMD metric.

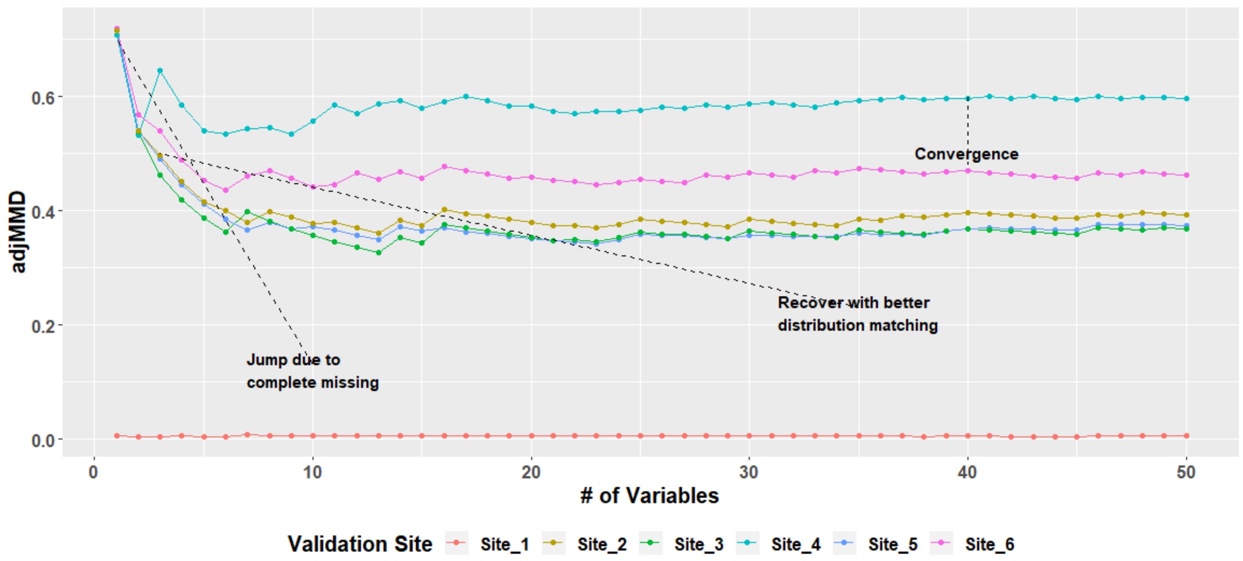

Supplemental Figure 21. Relationship between adjMMD and number of variables for model of 48-hour for moderate-to-severe AKI predictions with SCr and BUN removed, which shows the expected behaviors of adjMMD and identifies the top 13 variables to be a sufficient set to produce effective adjMMD metric.

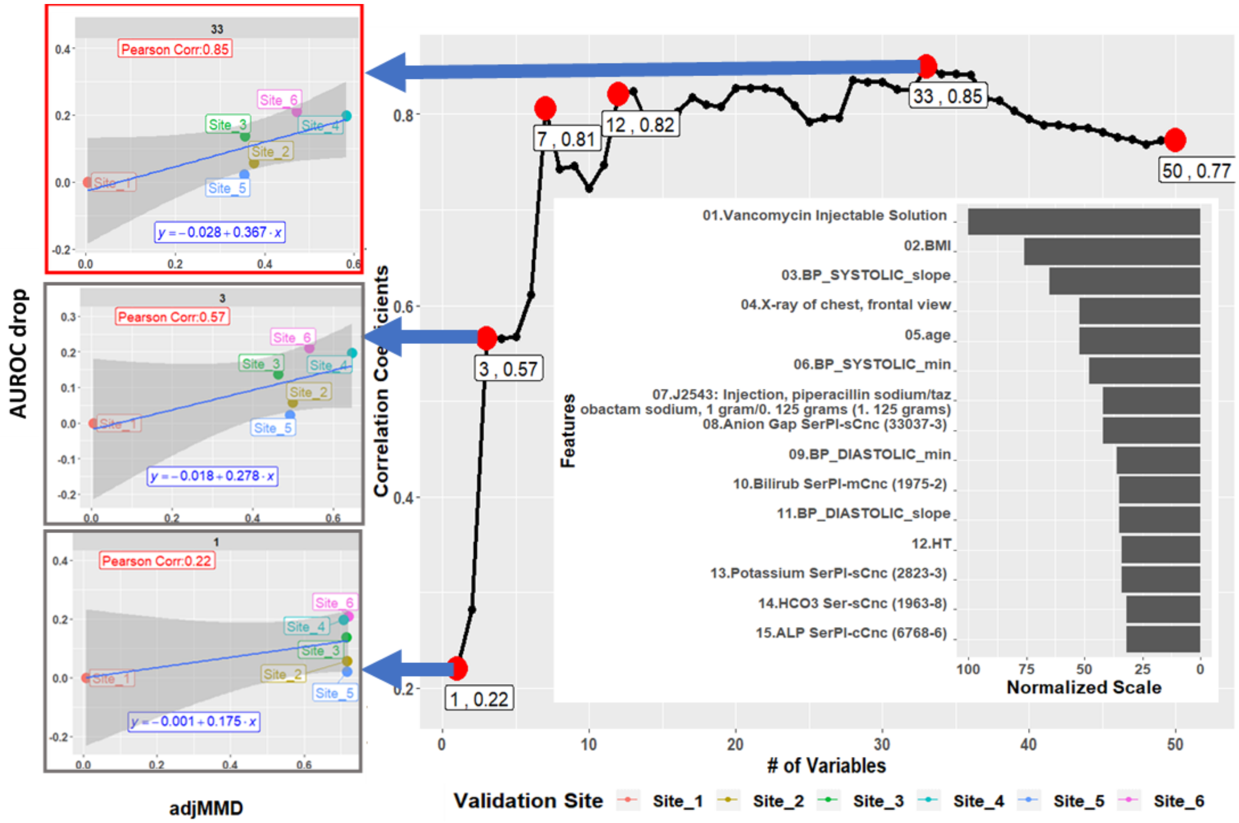

Supplemental Figure 22. Correlation Between adjMMD and Prediction Performance (AUC) for predicting moderate-to-severe AKI in 48 hours with SCr and BUN removed. Experimental results suggest that when top 33 important features are included for predicting moderate-to-severe AKI in 48 hours, the strength of association between adjMMD and AUC drops reaches an optimal value of 0.85 measured by Pearson correlation coefficient. The three panels on the left demonstrate the simple regression lines between adjMMD and AUC drop with 95% confidence band shaded.

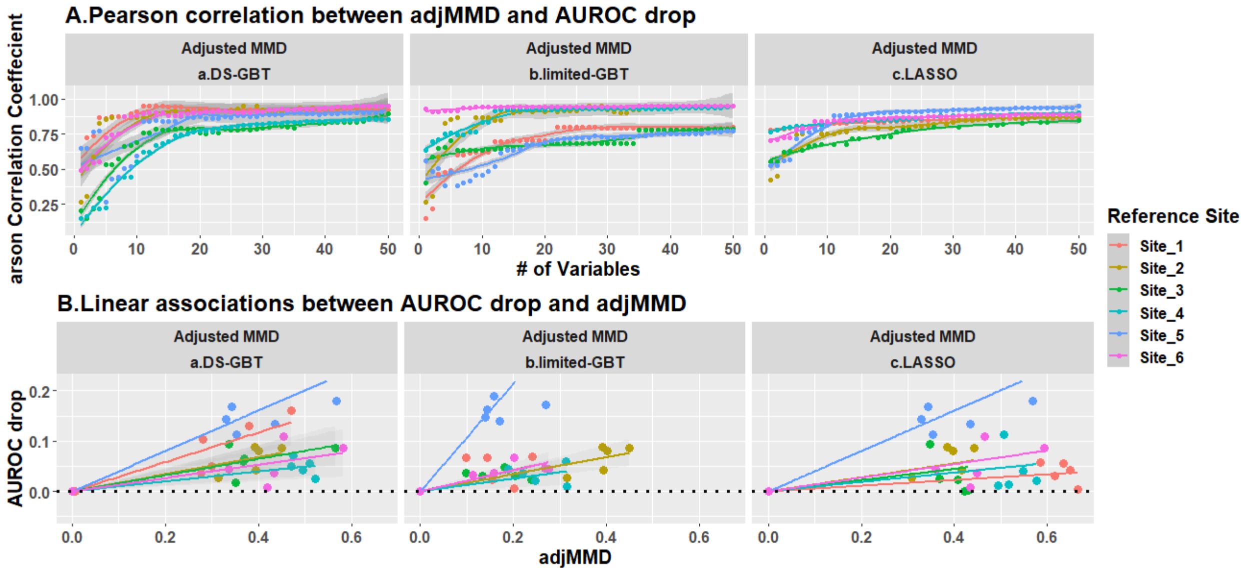

Supplemental Figure 23. Correlation Between adjMMD and Prediction Performance (AUC) for predicting moderate-to-severe AKI in 48 hours with varying models and derivation sites. Panel A shows the Pearson correlation coefficients between adjMMD and ΔAUC with respect to increasing feature size among the 3 experimental models (DS-GBT, limited-GBT, and LASSO) and varying derivation site. Panel B shows the positive linear relationship between adjMMD and ΔAUC for each model and derivation site.

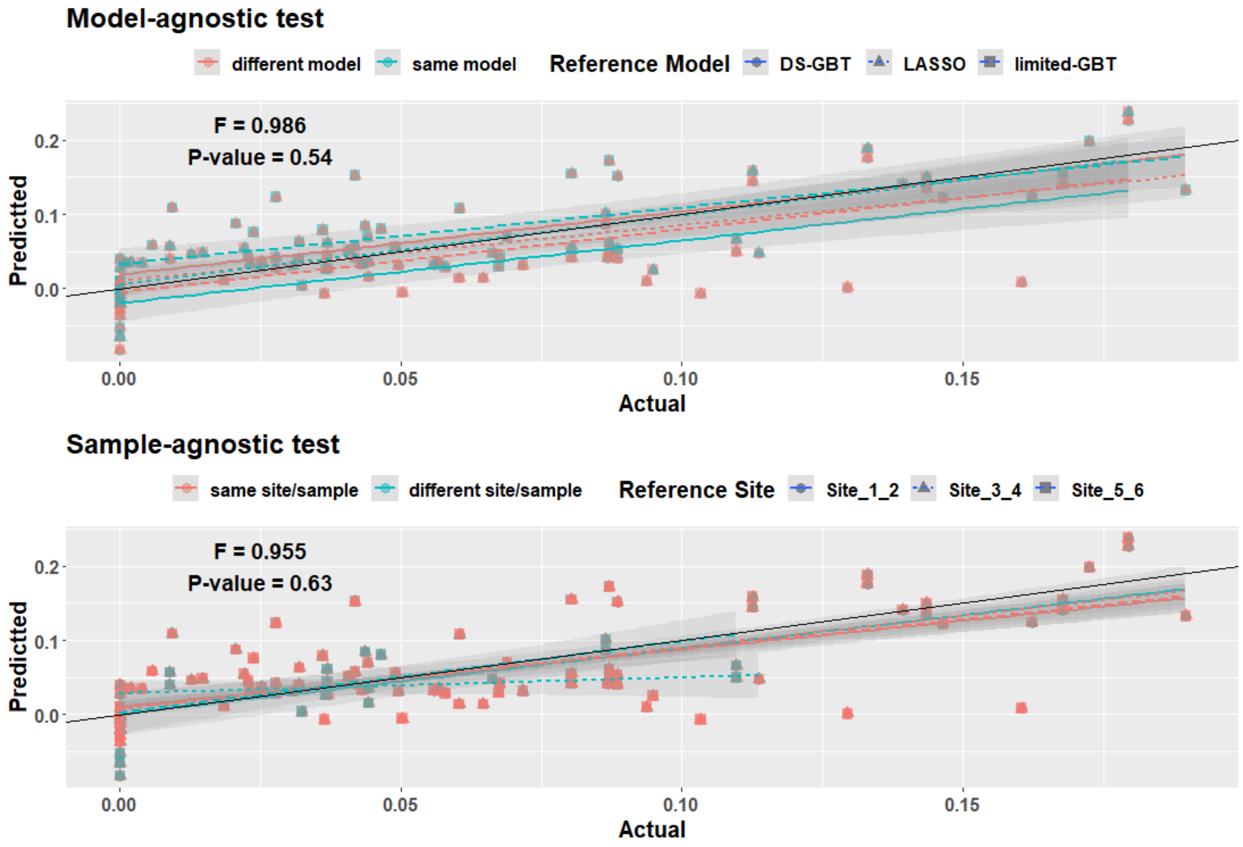

Supplemental Figure 24. Predicted vs. Actual plots demonstrating the Model-agnostic and Sample-agnostic test results. Panel A shows the result of Model-agnostic test. Each line in color represents a predicted vs. actual calibration for an adjMMD- ΔAUC linear regression model derived for one of the 3 experimental models (DS-GBT, limited-GBT, and LASSO). The green lines show "same-model" fitting results and the red lines the "different-model" fitting results. The F-score calculates the ratio of excessive residual sum of square (rss) for "different-model" over "same-model" (i.e. very small F-score suggests that the linear relationship between adjMMD and ΔAUC is generalizable across different models). Panel B demonstrates similar information as in A but for the Sample-agnostic test. The 'shaded areas' represent 95% confidence band for the calibration lines.

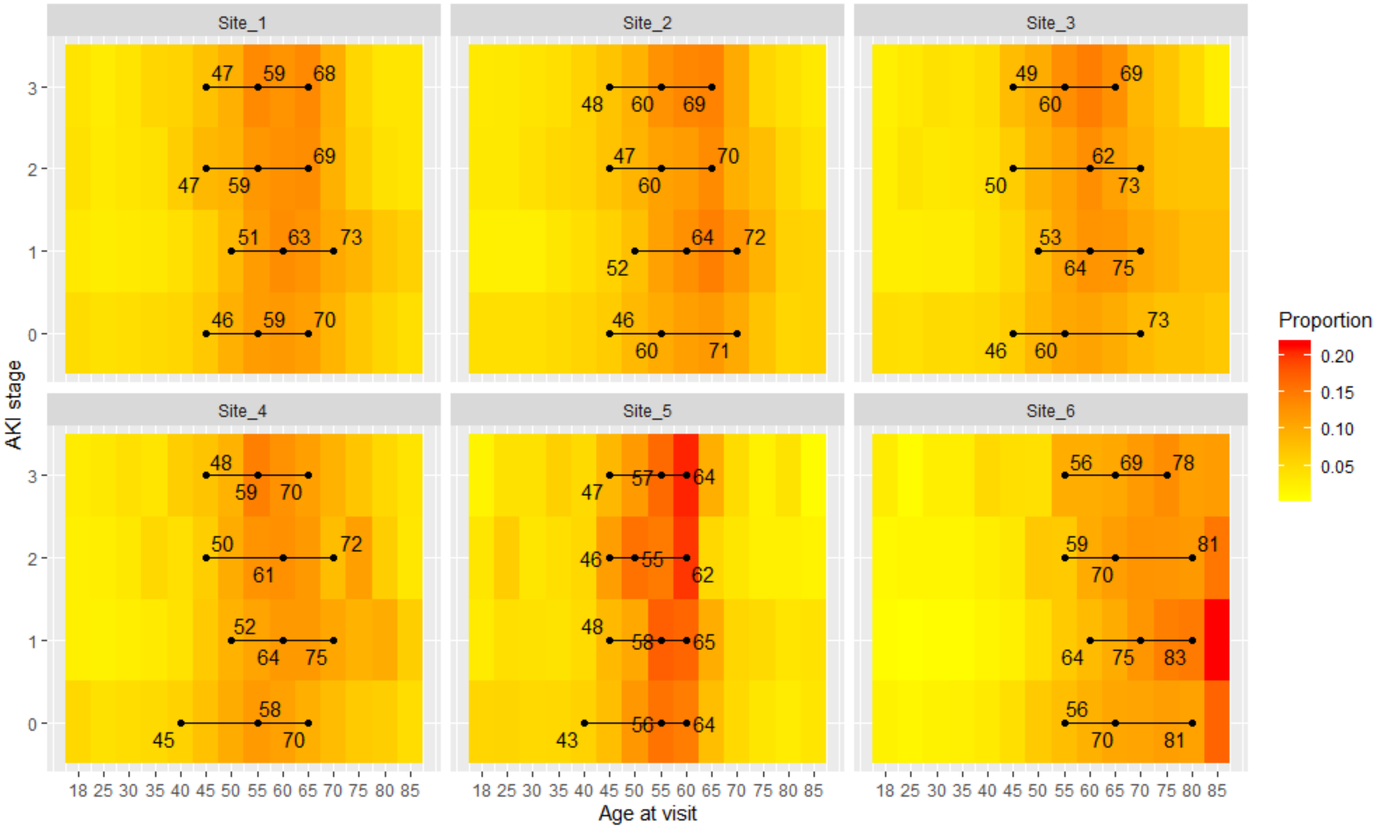

Supplemental Figure 25. Age distributions over different AKI stages across source and target health systems. Each vertical line showed the span of age from the 1st quartile to the 3rd quartile with median marked in the middle.

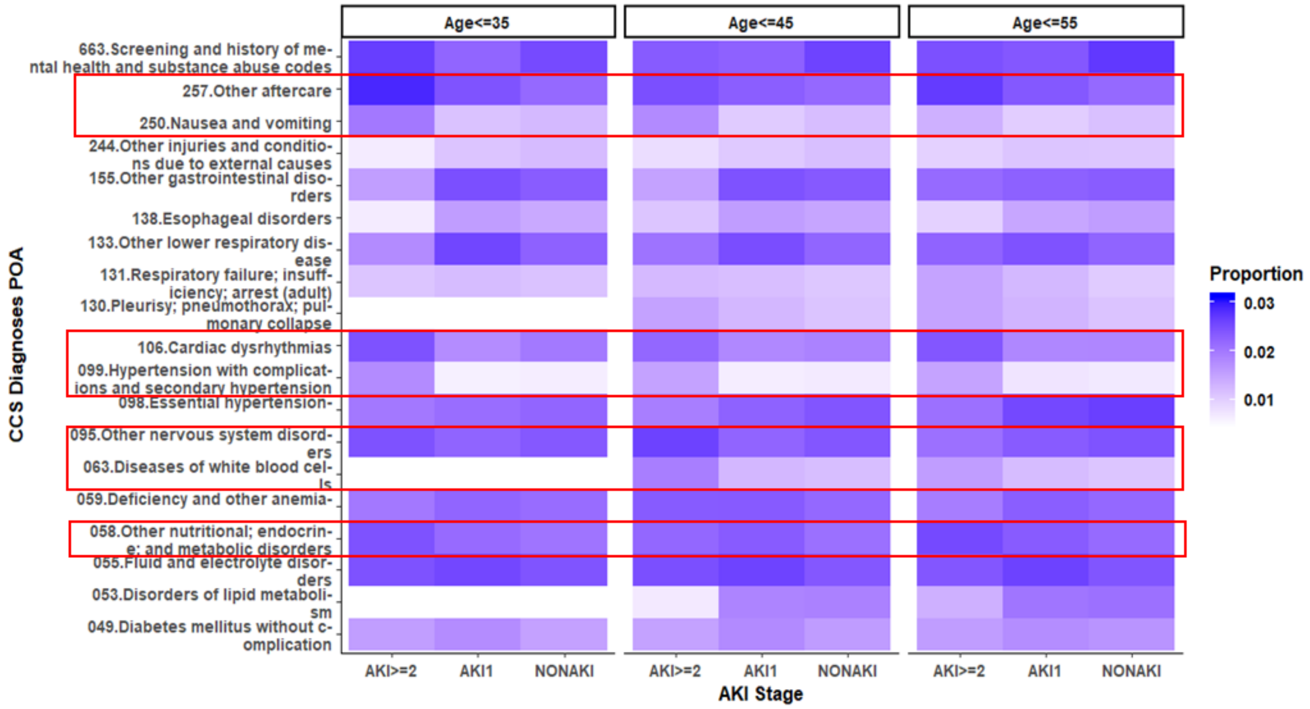

Supplemental Figure 26. Reason for admission for patients within different age group whom developed AKI of different AKI severity in the hospital.

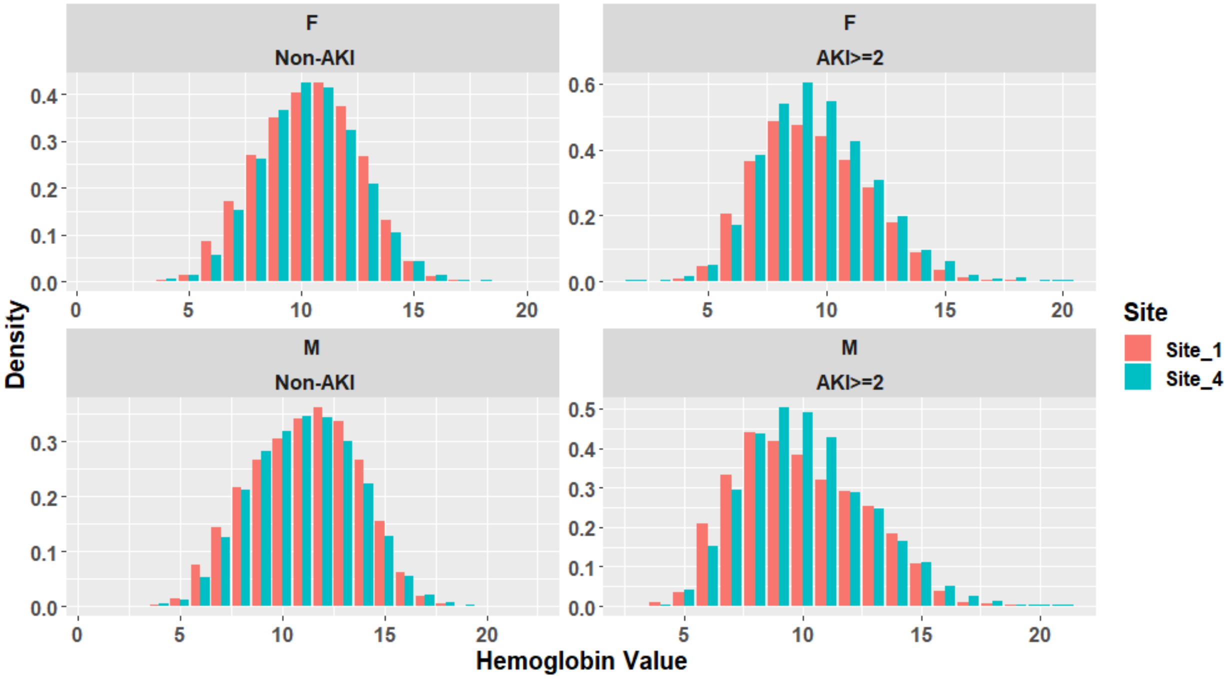

Supplemental Figure 27. Hemoglobin distribution comparisons between non-AKI patients and moderate-to-severe AKI for Site 1 and Site 4. Site 1 and Site 4 identified effects of hemoglobin with opposite directions (see Extended Figure 7).

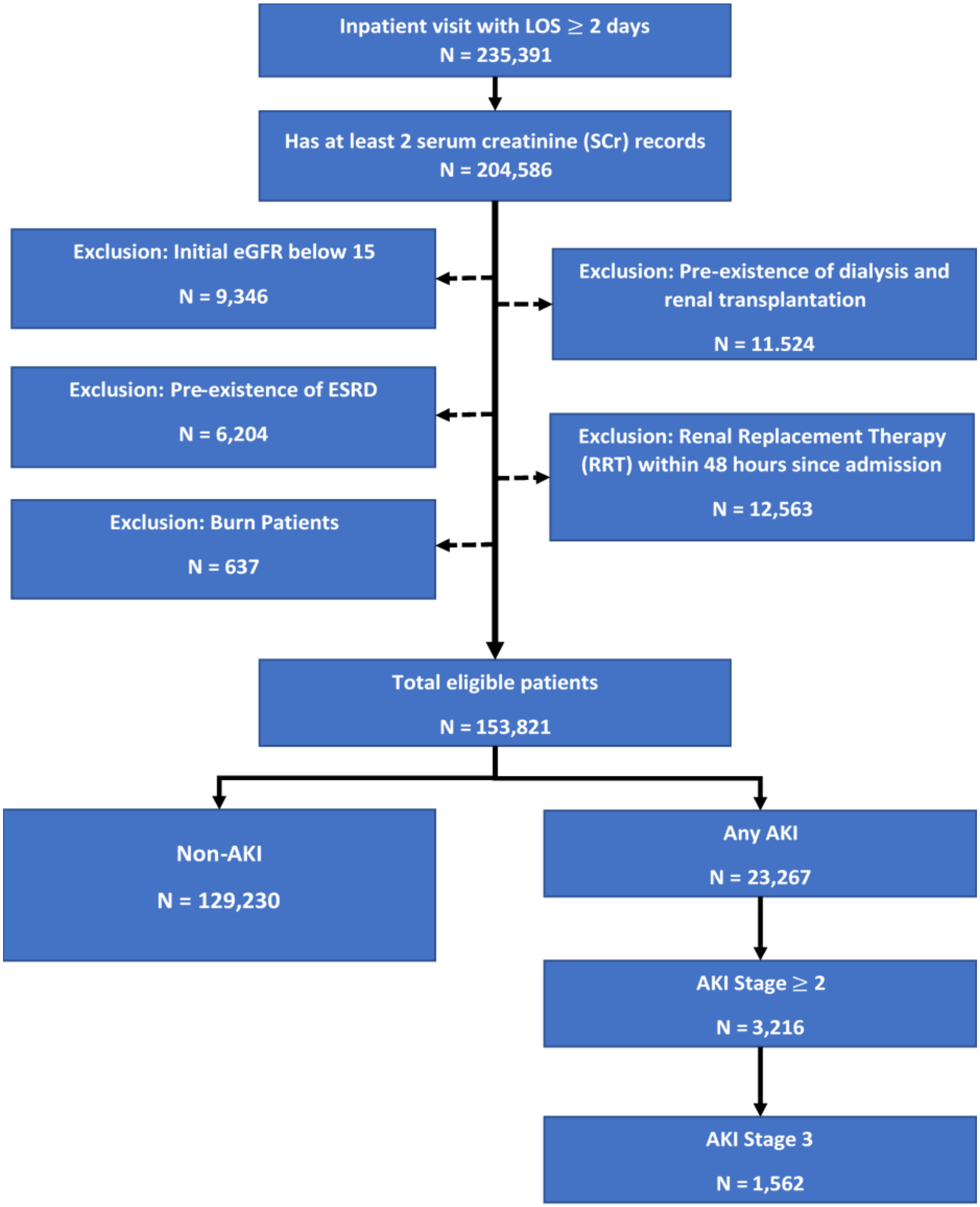

Supplemental Figure 28. Consort diagram for patient inclusions and exclusions.
