# Supplementary material for: Cross-site transportability of an explainable artificial intelligence model for acute kidney injury prediction
Source: Nat Commun. 2020 Nov 9;11:5668. doi: 10.1038/s41467-020-19551-w (PMC7653032; doi:10.1038/s41467-020-19551-w)
Supplement: Supplementary file 2 — Reporting Summary [file 41467_2020_19551_MOESM2_ESM.pdf]

nature research

Corresponding author(s): Mei Liu and Yong Hu
Last updated by author(s): Oct 14, 2020

Reporting Summary

Nature Research wishes to improve the reproducibility of the work that we publish. This form provides structure for consistency and transparency in reporting. For further information on Nature Research policies, see our Editorial Policies and the Editorial Policy Checklist.

Statistics

For all statistical analyses, confirm that the following items are present in the figure legend, table legend, main text, or Methods section.

n/a	Confirmed	
☐	☒	The exact sample size (n) for each experimental group/condition, given as a discrete number and unit of measurement
☐	☒	A statement on whether measurements were taken from distinct samples or whether the same sample was measured repeatedly
☐	☒	The statistical test(s) used AND whether they are one- or two-sided<br>*Only common tests should be described solely by name; describe more complex techniques in the Methods section.*
☐	☒	A description of all covariates tested
☐	☒	A description of any assumptions or corrections, such as tests of normality and adjustment for multiple comparisons
☐	☒	A full description of the statistical parameters including central tendency (e.g. means) or other basic estimates (e.g. regression coefficient) AND variation (e.g. standard deviation) or associated estimates of uncertainty (e.g. confidence intervals)
☐	☒	For null hypothesis testing, the test statistic (e.g. F, t, r) with confidence intervals, effect sizes, degrees of freedom and P value noted<br>*Give P values as exact values whenever suitable.*
☒	☐	For Bayesian analysis, information on the choice of priors and Markov chain Monte Carlo settings
☒	☐	For hierarchical and complex designs, identification of the appropriate level for tests and full reporting of outcomes
☐	☒	Estimates of effect sizes (e.g. Cohen's d, Pearson's r), indicating how they were calculated

Our web collection on statistics for biologists contains articles on many of the points above.

Software and code

Policy information about availability of computer code

Data collection	Data collection was performed by independent members of participating sites from Greater Plain Collaborative network without involvement from research team members. Collection was performed using institutional EHR systems (Epic or Cerner) and associated databases.
Data analysis	We used R/R studio (1.0.136) for data cleaning and analysis, the analysis codes can also be found in the github repo: https://github.com/kumc-bmi/AKI_CDM. The Gradient Boosting Tree-based Machines (GBT) algorithm was implemented using the R package xgboost

For manuscripts utilizing custom algorithms or software that are central to the research but not yet described in published literature, software must be made available to editors and reviewers. We strongly encourage code deposition in a community repository (e.g. GitHub). See the Nature Research guidelines for submitting code & software for further information.

Data

Policy information about availability of data

All manuscripts must include a data availability statement. This statement should provide the following information, where applicable:

- Accession codes, unique identifiers, or web links for publicly available datasets
- A list of figures that have associated raw data
- A description of any restrictions on data availability

The clinical data used for the training, validation and test sets were collected at individual participating site from Greater Plain Collaborative (GPC) data network and transferred to a secure data center with restricted access controls in de-identified format. The de-identified dataset may be available from the Greater Plains Collaborative clinical data network, subjective to individual institution’s and network-wide data governance and ethical approvals.

Field-specific reporting

Please select the one below that is the best fit for your research. If you are not sure, read the appropriate sections before making your selection.

- [x] Life sciences
- [ ] Behavioural & social sciences
- [ ] Ecological, evolutionary & environmental sciences

For a reference copy of the document with all sections, see nature.com/documents/nr-reporting-summary-flat.pdf

Life sciences study design

All studies must disclose on these points even when the disclosure is negative.

Sample size	No sample size calculation was performed prior to the study. The study cohort was defined using inclusion and exclusion criteria based on clinical expertise. This is a multi-center, retrospective, observational study with a total number of 506,597 eligible admissions for model development (site with the smallest sample still contains 19,542 admissions). This overall sample size is larger than most of similar studies. In addition, a sample size requirement of 200 patients would be required to detect sensitivity and specificity at 0.05 marginal error and 95% confidence. The smallest training sample still exceeded this requirement by two orders of magnitude.
Data exclusions	Patient exclusion criteria was pre-established. We excluded patients if they had evidence of severe kidney dysfunction at or before admission, that is a) estimated Glomerular Filtration Rate less than 15 mL/min/1.73m2, or b) has undergone any dialysis procedure or renal transplantation (RRT) prior to the visit, or c) required RRT within 48 hours of their first documented SCr measurement. Burn patients were also excluded since serum creatinine becomes a less reliable tool in assessing renal function during hypermetabolic phase.
Replication	Our prediction model was validated on clinical data from 6 health systems, that is using dataset from each health system as training and remaining sites for external validation.
Randomization	Sample allocation into derivation, calibration, and validation datasets are random.
Blinding	When randomly assigning patients to test, validation and training groups, investigators were blinded to patient covariates and all variables in the EHR not required to perform the research (e.g., serum creatinine was required to label AKI as a ground truth). Patient recruitment was conducted independently by participating sites of Greater Plain Collaborative data network. Research team members were blinded to this recruitment.

Reporting for specific materials, systems and methods

We require information from authors about some types of materials, experimental systems and methods used in many studies. Here, indicate whether each material, system or method listed is relevant to your study. If you are not sure if a list item applies to your research, read the appropriate section before selecting a response.

Materials & experimental systems

n/a	Involved in the study
x	Antibodies
x	Eukaryotic cell lines
x	Palaeontology and archaeology
x	Animals and other organisms
	x Human research participants
x	Clinical data
x	Dual use research of concern

Methods

n/a	Involved in the study
x	ChIP-seq
x	Flow cytometry
x	MRI-based neuroimaging

Human research participants

Policy information about studies involving human research participants

Population characteristics	The data included all patients from 6 participating GPC sites aged between 18 and 90 who were hospitalized for at least 2 days with at least two serum creatinine (SCr) records from the beginning of 2010 to the end of 2018. Patients were excluded if they had evidence of severe kidney dysfunction at or before admission, that is a) estimated Glomerular Filtration Rate less than 15 mL/min/1.73m2, or b) has undergone any dialysis procedure or renal transplantation (RRT) prior to the visit, or c) required RRT within 48 hours of their first documented SCr measurement. Burn patients were also excluded since serum creatinine becomes a less reliable tool in assessing renal function during hypermetabolic phase. Average age was 60 with males represented 51% of the test population. AKI occurred in 15.5% of admissions. Demographic profiles vary across participating GPC sites. More details on the demographic characteristics of the population at each institution are included in the manuscript (Table 1).
Recruitment	The clinical data assembled for this study were collected by the Greater Plain Collaborative (GPC), a Patient Centered Outcome Research Network (PCORnet) Clinical Data Research Network (CDRN) including twelve healthcare systems in nine states. No patients were excluded based on location, and no other exclusion criteria were applied. The final dataset consisted 506,597 eligible admissions that met inclusion and exclusion criteria. For more information please refer to the manuscript.
Ethics oversight	This study was determined not to be human subject research by the institutional review board of the GPC consortium because it only involved collection of existing and de-identified patient medical data.

Note that full information on the approval of the study protocol must also be provided in the manuscript.
